# Metformin Attenuates Ischemia-reperfusion Injury of Fatty Liver in Rats Through Inhibition of the TLR4/NF-κB Axis

**DOI:** 10.4274/balkanmedj.galenos.2020.2019.9.31

**Published:** 2020-06-01

**Authors:** Xiaohua Li, Liangliang Wang, Xiaoguang Yang, Chunyan Huang

**Affiliations:** 1 Department of General Surgery, Affiliated Hospital of Jiujiang University Jiangxi, China; 2 Department of Pathology, Affiliated Hospital of Jiujiang University, Jiangxi, China; 3 Department of Ultrasonic, Affiliated Hospital of Jiujiang University, Jiangxi, China; 4 Department of Hospitality, Affiliated Hospital of Jiujiang University, Jiangxi, China

**Keywords:** Fatty liver, inflammation, ischemia-reperfusion injury, liver transplantation, metformin

## Abstract

**Background:**

Donor organs for liver transplantation may often have fatty liver disease, which confers a higher sensitivity to ischemia/reperfusion injury. At present, there is no effective treatment for the condition. Evidence has suggested that metformin, the first-line medication for diabetes, has protective effects against many disorders. However, the potential role of metformin in ischemia/reperfusion injury in fatty liver disease remains unclear.

**Aims:**

To examine the effect of metformin treatment during ischemia/reperfusion injury in fatty liver and determine the possible mechanisms.

**Study Design:**

Animal experimentation.

**Methods:**

Sprague-Dawley male rats were fed a high-fat diet (520 kcal/100 g) for 14 weeks and then were subjected to the orthotopic autologous liver transplantation model. Sections of liver tissue were stained with hematoxylin and eosin to visualize the damage. Blood and liver samples were used to analyze the related proteins and components involved in the inflammatory signaling pathway.

**Results:**

We found that metformin significantly ameliorated the ischemia/reperfusion injury of the fatty liver through a reduction in alanine aminotransferase/aspartate aminotransferase concentrations in the serum and a decrease in dead cells, as shown by the terminal deoxynucleotidyl transferase-mediated dUTP nick-end labeling assay (p<0.05). In addition, metformin significantly attenuated interleukin (IL)-6, IL-1β, and tumor necrosis factor-α production and increased the expression of active caspase-3 and Bax in the liver (p<0.05). Mechanistically, metformin suppressed the activation of toll-like receptor 4 (TLR4)/NF-κB signaling (p<0.05), resulting in a decreased inflammatory response and apoptosis.

**Conclusion:**

Our findings demonstrated that metformin attenuated ischemia/reperfusion injury in fatty liver disease via the TLR4/NF-κB axis, suggesting that metformin could have potential therapeutic applications in ischemia/reperfusion injury associated with liver transplantation.

Liver transplantation is a revolutionary treatment for late-stage liver disorders, such as liver cancer and cirrhosis (1). Recently, owing to the unavailability of liver donors, fatty livers, a common condition in marginal livers, have been used for liver transplantation (2). However, it is disadvantageous to use fatty livers as donor organs as they have a higher production of reactive oxygen species (ROS) and a greater sensitivity to ischemia/reperfusion (I/R) injury compared with control livers (3). I/R injury leads to a lower graft survival rate and a longer hospitalization duration (4). Therefore, it is extremely important to identify novel interventions that will halt the progression of I/R injury during liver transplantation.

I/R injury in the liver causes a series of responses, including ROS generation, neutrophil infiltration, and cytokine release. Ultimately, it leads to the death of hepatocytes and endothelial cells (5,6). The toll-like receptor (TLR) family of proteins, particularly TLR4, is reported to mediate the molecular processes of deleterious effects during I/R injury (7,8). TLRs are commonly expressed in liver tissue, including in hepatocytes and hepatic stellate cells (9). Previous studies demonstrated that the stimulation of the TLR pathway results in Nuclear Factor kappa B (NF-κB) activation and subsequently increases the protein expression of proinflammatory factors (10,11). Drugs that suppress the activation of the TLR pathway offer potential benefits in liver transplantation, similar to the effects seen after transgenic methods that block NF-κB- or TLR4-related genes (12,13,14).

Metformin, a biguanide used widely for the treatment of diabetes, reduces glucose production in the liver and increases the sensitivity of the liver and surrounding tissues to insulin (15,16). Thus, metformin has been suggested as a potential drug for multiple diseases, including cancers (17), cardiovascular diseases (18), diabetes (19), Huntington’s disease (20), and Alzheimer’s disease (21). However, the role of metformin in I/R injury in fatty liver has not yet been described. Therefore, we examined the effect of metformin treatment during I/R injury in fatty liver and determined the possible mechanisms.

## MATERIALS AND METHODS

### Surgical procedures

Experiments were conducted on Sprague-Dawley male rats (200-250 g), which were supplied by the Affiliated Hospital of Jiujiang University and housed in a pathogen-free environment. Every procedure was approved by the Animal Care and Use Committee of the Affiliated Hospital of Jiujiang University (date: 15/7/2019).

To induce steatosis, rats were fed a high-fat diet (520 kcal/100 g) for 14 weeks, comprising 60% fat, 20% carbohydrates, and 20% protein D12492. Rats were injected intraperitoneally with metformin at a dose of 50 mg/kg/day for 3 days until the rats were killed.

The following procedure was used to establish the orthotopic autologous liver transplantation (OALT) model. Under general anesthesia, the bile ducts, vessels, and ligaments surrounding the liver were dissociated carefully to expose the entire liver. Four vessels, namely, the portal vein (PV), super hepatic vena cava (SHVC), hepatic artery (HA), and inferior hepatic vena cava (IHVC), were dissected. Before the blockage of these blood vessels, 50 U of heparin saline solution was injected via the tail vein. Following the occlusion of the PV and HA, blood flow to the IHVC and SHVC was shut off. The liver was slowly (2.0 mL/min) perfused with 4°C heparin through the PV, and an outflow tract was made via a 1.0 mm hole in the IHVC. The allowed duration of ischemia was 20 min. The rats used in this study were euthanized after 8 h of reperfusion for the collection of blood and liver samples.

### Histopathological examination

The liver tissues collected for histopathological examination were fixed in 10% formalin and then cut into 4 µm slices. The percentage of necrotic area in steatotic livers was detected using hematoxylin and eosin staining.

### Terminal deoxynucleotidyl transferase-mediated dUTP nick-end labeling assay (TUNEL) staining

Apoptotic cells in the liver were detected by TUNEL staining following the manufacturer’s protocol (Roche, Germany). In brief, the liver samples were cut into 4 µm slices, pretreated with proteinase K, and incubated with TUNEL reagents at 37°C for 1 h. The liver slices were viewed at 400× magnification, and the TUNEL-positive dead cells were counted from five randomly selected fields in each slice.

### Serum alanine aminotransferase (ALT) and aspartate aminotransferase (AST) enzyme activity

Whole blood samples were collected and kept at room temperature for 40 min. The serum portion was isolated from whole blood by centrifugation at 4°C for 20 min at 1800 × *g*. The serum samples were then analyzed for ALT and AST activities using assay kits purchased from Fujifilm (Tokyo, Japan) and detected using a DRI-CHEM 3500i biochemical analyzer (Fujifilm) following the vendor’s instructions.

### Enzyme‑linked immunosorbent assay (ELISA)

The liver tissue samples were cut into small pieces, homogenized in lysis buffer, and centrifuged at 4°C for 20 min at 12000 × *g*. ELISA kits purchased from Keygen Biotech (Nanjing, China) were used to determine the concentration of the proteins of interest [tumor necrosis factor (TNF)-α, interleukin (IL)-10, IL-1β, and IL-6] in the liver samples.

### Catalase (CAT) and superoxide dismutase (SOD) activities

The liver tissue samples were homogenized and centrifuged at 4°C for 20 min at 12000 × g. The CAT and SOD activities in the liver samples were measured using commercially available kits (Jianchen Biotech, Nanjing, China).

### Western blotting analysis

The liver tissues for Western blotting were homogenized, and the total protein concentration was calculated using a Bradford assay (BioRad, CA, USA). A standard sodium dodecyl sulfate-polyacrylamide gel electrophoresis procedure was used to analyze the protein samples; the proteins were separated on a premade 8-15% Tris-HCl polyacrylamide protein gel (BioRad) and then transferred onto 0.45 µm polyvinylidene fluoride (PVDF) membranes (Millipore, USA). Nonspecific binding to the membranes was blocked, and the membranes were incubated individually with antibodies against fibronectin, TLR4, p-p65, p65, p-IKK, IKK, Bax, Bcl-2, caspase-3, and β-actin in Tris-buffered saline-0.1% Tween buffer in a refrigerator overnight. The PVDF membranes were exposed to horseradish peroxidase-conjugated antibodies and then analyzed by the application of enhanced chemiluminescence reagent (Pierce, USA) using chemiluminescence imaging equipment (Omega 16ic, Ultra-Lum, Claremont, CA, USA).

### Statistical analysis

The data were presented as the mean ± standard deviation. One-way analysis of variance methods and Tukey’s post-hoc analysis were used to test the significance of the difference between multiple groups. P values of < 0.05 indicated a significant difference.

## RESULTS

### Metformin reduces I/R injury in steatotic livers in a mouse model of OALT

**Figure 1:**
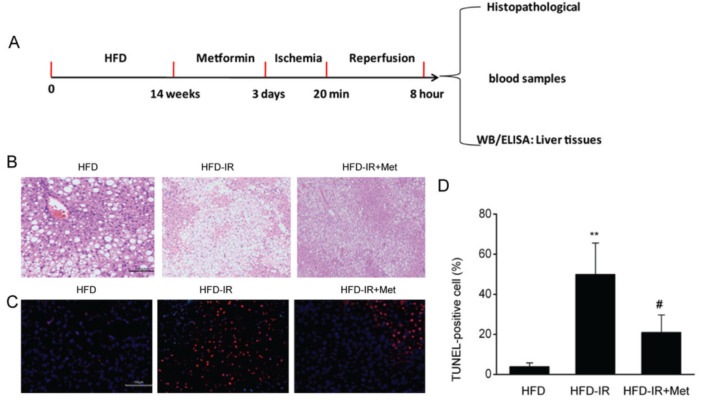
(A) Metformin reduces ischemia/reperfusion (I/R) damage in fatty liver in a rat model of orthotopic autologous liver transplantation. Flow chart. (B) Representative hematoxylin and eosin staining of sections of the liver of high fat diet (HFD)-fed rats after the induction of I/R injury and after metformin treatment. Scale bars =200 μm. (C) Representative terminal deoxynucleotidyl transferase-mediated dUTP nick-end labeling assay (TUNEL) staining. Scale bars =100 μm. (D) Quantitative analysis of data in B (8-10 sections per rat were analyzed). The results are shown as the mean ± standard deviation, sample number, n=3, One-Way analysis of variance. **P-value <0.01 compared with the HFD group, ^#^p-value <0.05 compared with the HFD-I/R group. (This definition of the significance value of p applies to all the figures). ELISA: enzyme-linked immunosorbent assay, WB: western blot

Steatotic livers that underwent OALT (Figure 1A) displayed lobular distortion, severe edema, sinusoidal congestion, inflammatory cell infiltration, and necrosis (greater than 70%). Pretreatment with metformin reduced the histological damage in the I/R-injured livers (Figure 1B). In addition, the number of apoptotic cells was increased in the I/R-injured livers compared with that in control fatty livers. Metformin pretreatment considerably reduced apoptotic cells in the I/R-injured livers (Figure 1C, D). These data indicated that metformin protected fatty livers from I/R damage.

### Metformin improves the function of steatotic livers subjected to I/R injury

**Figure 2:**
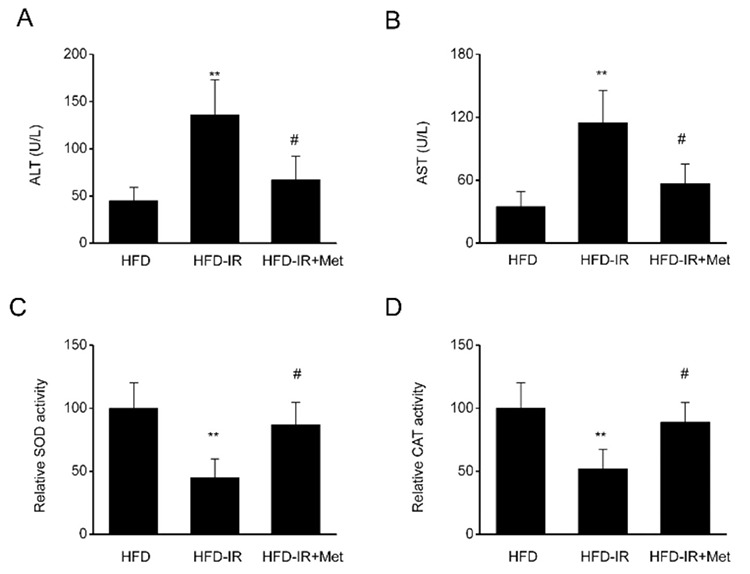
Metformin improves liver function in steatotic livers subjected to ischemia/reperfusion (I/R) injury. Serum concentrations of (A) alanine transaminase (ALT) / (B) aspartate transaminase (AST) in high-fat diet (HFD)-fed rats after the induction of I/R injury and after metformin treatment. The activity of (C) superoxide dismutase (SOD) and (D) catalase (CAT) was calculated in the HFD-fed rats after the induction of I/R injury and metformin treatment. N=8, One-Way analysis of variance. **P-value <0.01 compared with the HFD group, ^#^p-value <0.05 compared with the HFD-I/R group.

AST and ALT levels were used as markers of liver injury. We found that the AST and ALT levels were elevated in I/R-injured steatotic livers and that these levels were significantly decreased by metformin treatment (Figure 2A, B). CAT and SOD play essential roles in ischemic injury and antioxidative effects. As shown in Figure 2C and D, the activity of CAT and SOD was considerably upregulated in the I/R injury in fatty liver, and this effect was reversed by metformin treatment.

### Metformin reduces the release of cytokines in I/R injury of fatty liver

**Figure 3:**
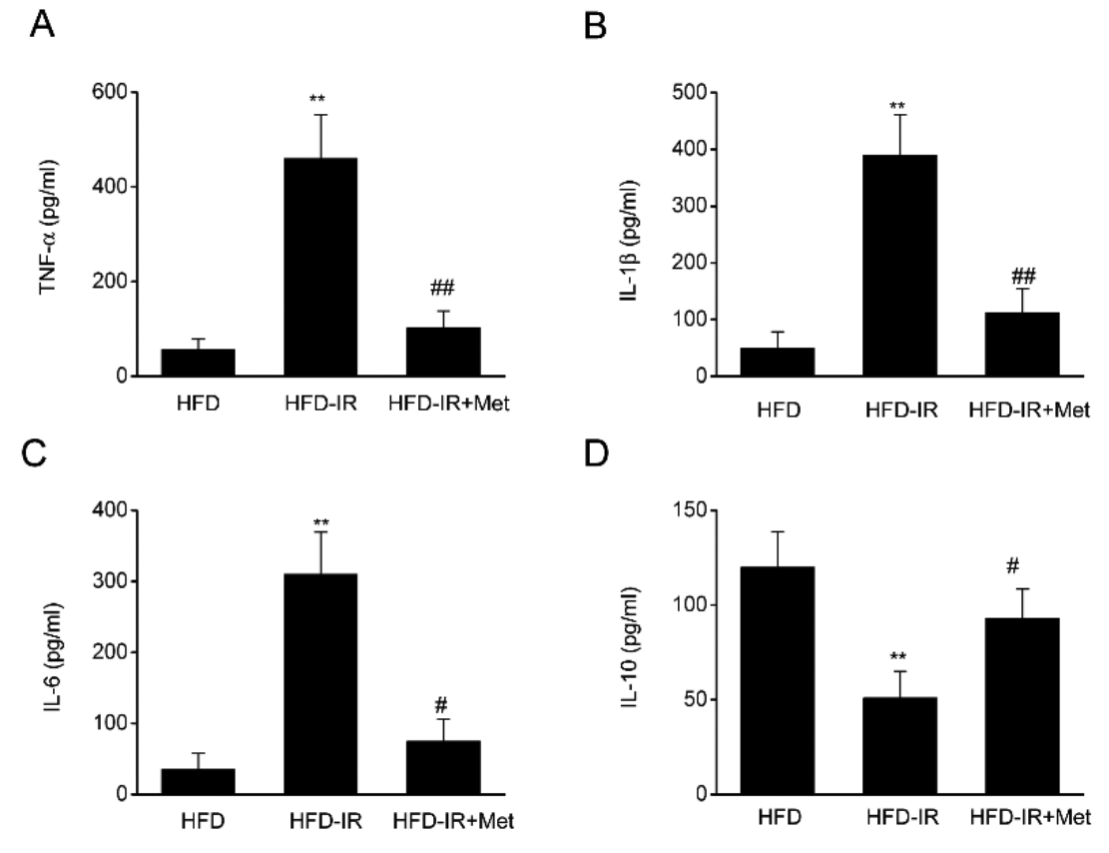
Metformin reduces the release of cytokines in ischemia/reperfusion (I/R)-injured fatty liver. (A) Tumor necrosis factor (TNF)-α, (B) interleukin (IL)-1β, (C) IL-6, and (D) IL-10 expression in high-fat diet (HFD)-fed rats after the induction of I/R injury and metformin treatment. N=8, One-Way analysis of variance. **P-value <0.01 compared with the HFD group, ^#^p-value <0.05 compared with the HFD-I/R group.

To detect whether metformin affected the inflammatory response *in vivo*, we detected the expression of TNF-α and several interleukin family members in the liver. In Figure 3, the protein expression of TNF-α and IL family members was markedly upregulated in groups subjected to I/R injury, but this was markedly ameliorated by metformin treatment. Meanwhile, the expression of IL-10 was considerably decreased in the I/R-injured steatotic livers, and this effect was markedly reversed by metformin.

### Metformin inhibits TLR4/NF-κB signaling in the I/R injury of steatotic livers

**Figure 4:**
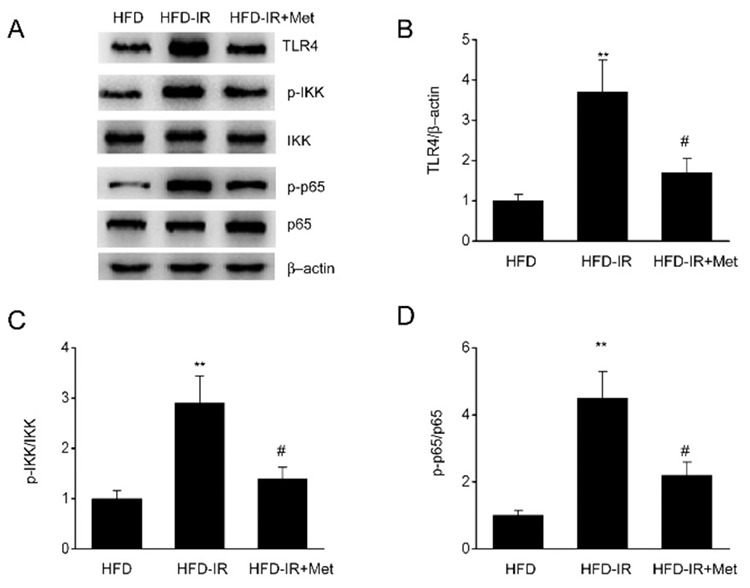
Metformin inhibits toll-like receptor (TLR)4/NF-κB signaling in ischemia/reperfusion (I/R) injury of the steatotic liver. Representative (A) immunoblotting and (B) TLR4, (C) p-IKK, and (D) p-p65 analysis in high-fat diet (HFD)-fed rats after I/R injury and metformin treatment. N=3, One-Way analysis of variance. **P-value <0.01 compared with the HFD group, ^#^p-value <0.05 compared with the HFD-I/R group.

The TLR4/NF-κB pathway has a key role in the biogenesis of cytokines and ischemic injury (22). As shown in Figure 4, the expression of phosphorylated NF-κB, p65, TLR4, and phosphorylated IKK was significantly upregulated in the steatotic liver after I/R injury. However, metformin treatment largely reversed the expression of these components in the TLR4/NF-κB pathway. These findings indicated that metformin reduced inflammation via the inhibition of TLR4/NF-κB signal activation in I/R-injured steatotic livers.

### Metformin suppresses apoptosis-related protein expression in I/R-injured steatotic liver

**Figure 5:**
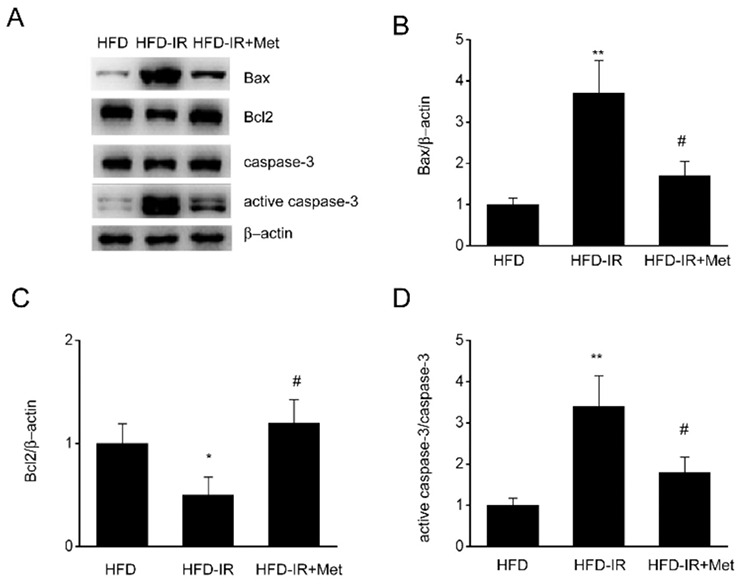
Metformin suppresses apoptosis-related proteins expression in ischemia/reperfusion (I/R)-injured steatotic liver. Representative (A) immunoblots and quantitative analysis of (B) Bax, (C) Bcl-2, and (D) active caspase-3 in high-fat diet (HFD)-fed rats after I/R and metformin treatment. N=3, One-Way analysis of variance. **P-value <0.01 compared with the HFD group, ^#^p-value <0.05 compared with the HFD-I/R group.

It is known that inflammation induces cell apoptosis and that apoptosis-related proteins play an important role in cell apoptosis (23). We then examined whether metformin affected the expression of apoptosis-related proteins. We found that the expression of Bcl-2 was decreased and that the expression of active caspase-3 and Bax was markedly increased in the I/R-injured steatotic liver. However, this could not be observed with metformin pretreatment (Figure 5). Our results indicated that metformin reduced apoptosis via the regulation of the expression of apoptosis-related proteins.

## DISCUSSION

Over the past 20 years, lifestyles have changed extensively. A prominent feature of these new lifestyles is overeating and physical inactivity, which are the most common causes of obesity and nonalcoholic fatty liver disease (NAFLD). Currently, the global occurrence of obesity and NAFLD is approximately 24% (24), which increases the risk of problems, such as steatotic livers, with donor organs from affected individuals (25). However, one of the most common disadvantages of fatty liver transfer is the risk of I/R injury, which has been confirmed in both animal experiments and clinical studies. At present, no effective method has been developed to alleviate this risk. Therefore, more research should be conducted to prevent I/R after fatty liver transplantation. The mechanism of NAFLD is well explained by the traditional two-hit theory. The “first hit” is hepatic steatosis, and the “second hit” includes inflammatory mediators and increased ROS generation (26). Our study investigated whether metformin had ameliorative effects on I/R injury after steatotic liver transplantation. Using our rat model of OALT, we found that metformin suppressed TLR4/NF-κB signaling, decreased the extent of inflammatory factors, and impaired hepatic injury induced by OALT.

Several molecular mechanisms have been proposed for metformin’s mode of action, such as inhibition of mTOR, inhibition of mitochondrial complex-1, or replenishing hepatocyte glutathione. Our study found that metformin decreased TLR4/NF-κB signaling through downregulation of the expression TLR4, p65, and IKK in the liver and inhibition of the release of the cytokines before inflammation. Previous studies have shown that endogenous ligands, such as damage-associated molecular patterns and cytokines, triggered TLR4 (27). NF-κB promoted the production of inflammatory cytokines and then amplified the inflammatory responses (28). TLR4 activates NF-κB and is a key molecule in the mechanism of liver I/R injury (29,30). The extent of liver I/R injury was reduced in the absence of the TLR4 gene in the liver, and I/R injury was ameliorated in Kupffer cells and hepatocytes through the suppression of cytokine release before inflammation when TLR-4 was blocked (31). Some drugs block TLR4/NF-κB, resulting in anti-inflammatory and hepatoprotective effects; however, these pharmacologic agents are not routinely used in clinics or applied in liver transplantation (32,33). Collectively, our results suggested that metformin attenuated OALT-induced inflammation via the suppression of the TLR4/NF-κB signaling pathway.

Necrosis and cell death are the most common reactions of the liver injured by ischemia, radiation, and toxic substances (34). Hepatic parenchymal cells are sensitive to I/R-induced damage (35). I/R damage to enzymes and anti-apoptotic proteins is caused by oxidative factors and increases apoptosis. I/R-induced liver injury was notably attenuated by the inhibition of the caspase family, which indicated the important role of apoptosis in I/R injury (36). Hepatocyte cell death resulted in the upregulation of ALT and AST, which further reduced liver function (37). The use of TUNEL staining to elucidate hepatocellular apoptosis induced by I/R injury supported this observation. Consistent with other reports, our results showed that the percentage of hepatocyte cell death was considerably increased after I/R injury and that pretreatment with metformin reversed the situation. The AST and ALT levels were depressed in I/R-injured rats pretreated with metformin, which indicated that metformin was a key attenuator of the hepatocyte apoptosis induced by I/R. Previous studies have shown that apoptosis-related factors, Bax and Bcl-2, are involved in the regulation of apoptosis (38). In our study, we detected marked upregulation of the Bax protein, whereas that of Bcl-2 was considerably decreased. Metformin treatment effectively decreased Bax expression, which was associated with cleaved caspase-3 activation. Simultaneously, metformin treatment also promoted Bcl-2 expression. Our results suggest that the rescue effects of metformin treatment against I/R were achieved by blocking the regulators involved in programmed cell death.

However, it remains unclear how metformin inhibits the TLR4/NF-κB signaling pathway. In addition, the molecular mechanism of downregulation of apoptosis by metformin still needs further investigation. Future clinical studies are needed to evaluate the effect of metformin on I/R injury among patients with or without fatty liver.

In conclusion, our data demonstrate that metformin protects against I/R injury in liver organ transplantation through the inhibition of the TLR4/NF-κB axis, which implies that metformin has a potential therapeutic application in I/R injury induced by fatty liver transplantation.
